# Supplementary material for: Cold-induced FOXO1 nuclear transport aids cold survival and tissue storage
Source: Nat Commun. 2024 Apr 3;15:2859. doi: 10.1038/s41467-024-47095-w (PMC10991392; doi:10.1038/s41467-024-47095-w)
Supplement: Supplementary file 1 — Supplementary Information [file 41467_2024_47095_MOESM1_ESM.pdf]

Supplementary Information for

Cold-induced FOXO1 nuclear transport aids cold survival and tissue storage

Xiaomei Zhang[1,2,3*], Lihao Ge[4*], Guanghui Jin[1,2,5*], Yasong Liu[1*], Qingfen Yu[6*], Weizhao Chen[1,2], Liang Chen[1,2], Tao Dong[1,7], Kiyoharu J. Miyagishima[8], Juan Shen[2,9], Jinghong Yang[1,2], Guo Lv[9], Yan Xu[10], Qing Yang[1], Linsen Ye[1,2], Shuhong Yi[1], Hua Li[1], Qi Zhang[2,9,10], Guihua Chen[1,2,9], Wei Liu[2,9†], Yang Yang[1,2,9†], Wei Li[8†], Jingxing Ou[1,2,9†]

1 Department of Hepatic Surgery and Liver transplantation Center of the Third Affiliated Hospital, Organ Transplantation Institute, Sun Yat-sen University, Guangzhou, China

2 Guangdong Key Laboratory of Liver Disease Research, the Third Affiliated Hospital of Sun Yat-sen University, Guangzhou, China

3 Department of Cancer Biology, Dana-Farber Cancer Institute; Department of Cell Biology, Harvard Medical School, Boston, MA, USA

4 Institute of Psychiatry and Neuroscience, Xinxiang Medical University, Xinxiang, China

5 State Key Laboratory of Respiratory Disease, National Clinical Research Center for Respiratory Disease, Guangzhou Institute of Respiratory Health, The First Affiliated Hospital of Guangzhou Medical University, Guangzhou, China

6 Department of Neurology, The Third Affiliated Hospital of Sun Yat-sen University, Guangzhou, China

7 Department of Surgery, University of Michigan, Ann Arbor, MI, USA

8 Retinal Neurophysiology Section, National Eye Institute, National Institutes of Health, Bethesda, MD, USA

9 Guangdong province engineering laboratory for transplantation medicine, Guangzhou, China

10 Cell-gene Therapy Translational Medicine Research Center, the Third Affiliated Hospital of Sun Yat-sen University, Guangzhou, China

* Equal contributions.

† Correspondence to: WLIU lwei6@sysu.edu.cn YY yysysu@163.com

WLI liwei2@nei.nih.gov JO oujx7@sysu.edu.cn

Supplementary figures:

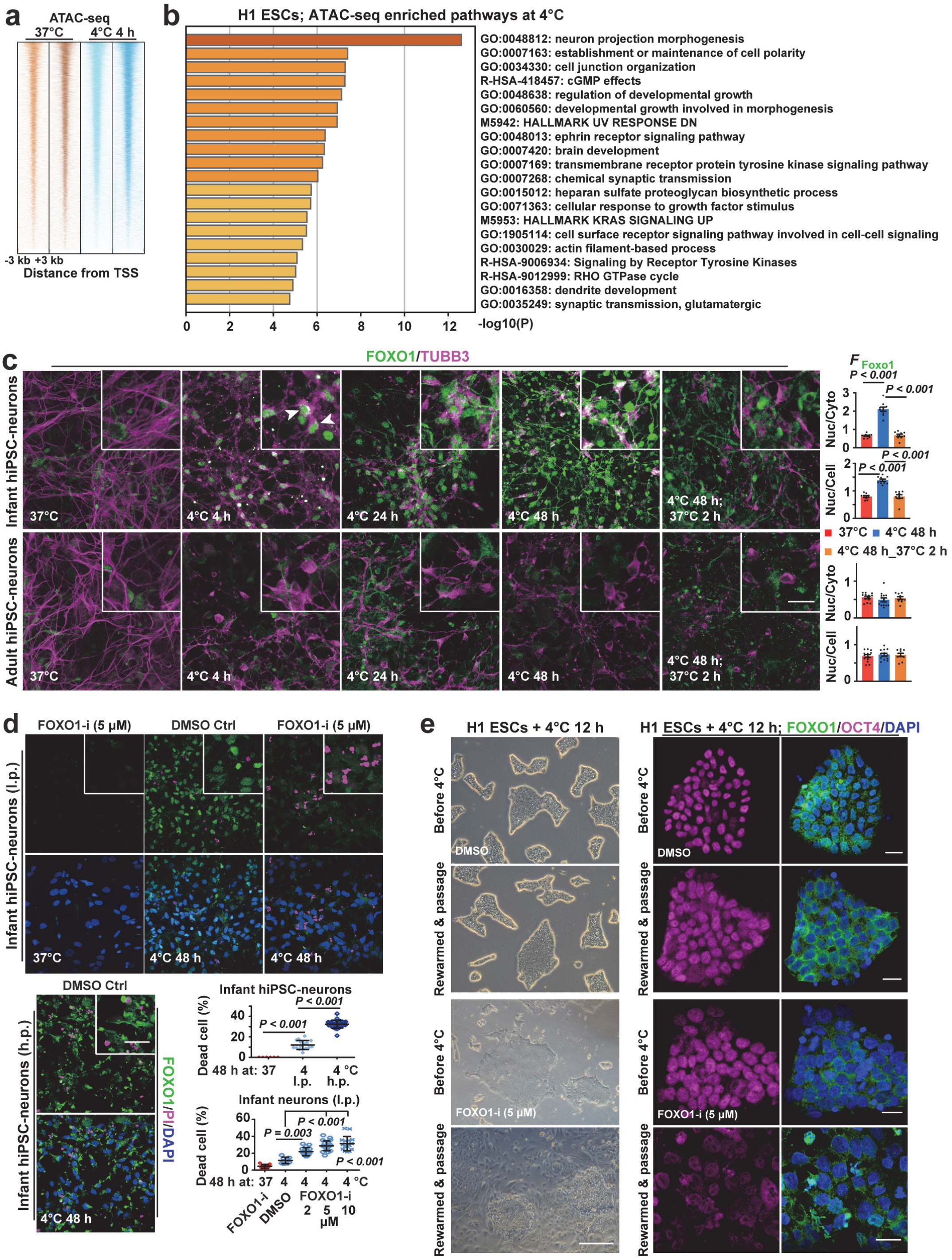

Supplementary Fig. 1 | Temperature-induced FOXO1 Subcellular Translocation in Human Cells. a, Heatmap of ATAC-seq signal intensity within 3 kb up- and down- stream of TSS (Transcription start site) in different samples; kb, kilobase pairs. **b**, Pathway enrichment analysis of genes highly accessible to transcription factor binding at 4°C. **c**, Left: confocal images of FOXO1 and TUBB3 staining in iPSC-

neurons derived from an infant donor and an adult donor; right: statistics of FOXO1 fluorescence (F_{FOXO1}) at indicated conditions (from left to right, infant iPSC-neurons: n = 10, 10 and 9 images; adult iPSC-neurons: n = 13, 13 and 8 images from 5 experiments). **d**, Left: confocal images of FOXO1, Propidium Iodide (PI; to stain dead cells) and DAPI signals in low-passage (l.p.) and high-passage (h.p.) infant iPSC-neurons; right: statistics on cell death in infant iPSC-neurons at indicated conditions (from left and right, top to bottom, n = 6, 16, 26, 8, 8, 16, 13 and 16 images from 5 experiments); FOXO1-i, FOXO1 inhibitor AS1842856. **e**, Left: light microscopy of H1 ESC colony morphology at indicated conditions; right: confocal images of FOXO1 and OCT4 staining in cultures of H1 ESCs at indicated conditions (n = 3 experiments). Data are shown as mean and SEM. Statistics: *P* values in (**b**) were calculated in Metascape; one-way ANOVA followed by Tukey's test (**c** and **d**). Scale bars: 20 µm (**c, d** and **e**: right), and 400 µm (**e**: left). Source data are provided as a Source Data file.

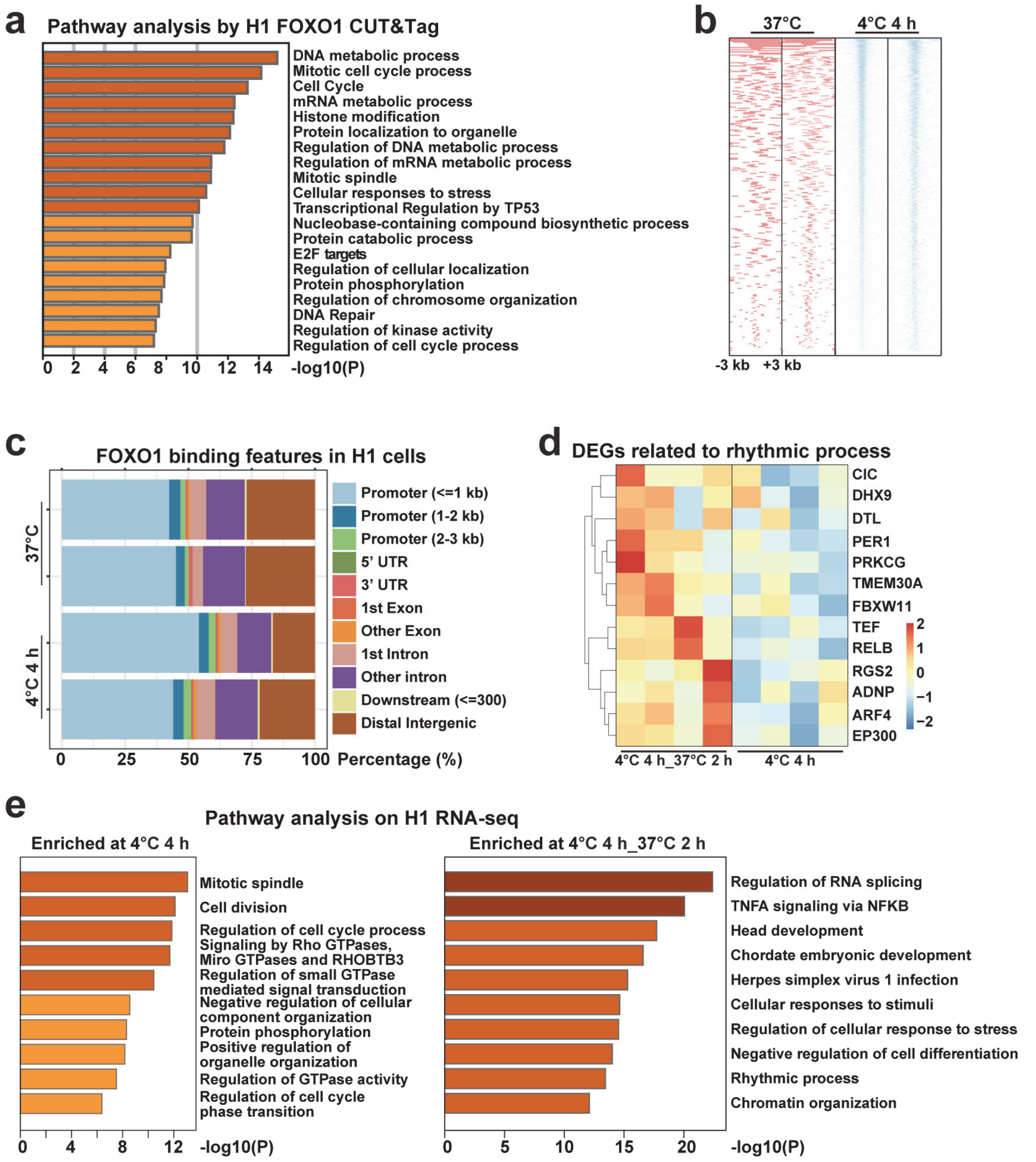

Supplementary Fig. 2 | CUT&Tag and Bulk-RNA-Seq analyses. a, Pathway enrichment analysis of the 1389 genes identified by CUT&Tag with enhanced FOXO1 binding at 4°C in H1 ESCs. **b**, Heatmap showing enhanced FOXO1 binding at 4°C in H1 cells within 3 kb up- and down-stream of TSS (Transcription start site). **c**, Stacked bar plots showing genomic annotation of FOXO1 binding regions in different groups. **d**, Heatmap showing the expression of DEGs related to rhythmic process during rewarming; the value for each gene is row-scaled Z score. **e**, Pathway enrichment analysis of the 2742 differentially expressed genes down-regulated (left) or up-regulated (right) during rewarming in H1 ESCs. *P* values in (**a** and **e**) were calculated in Metascape.

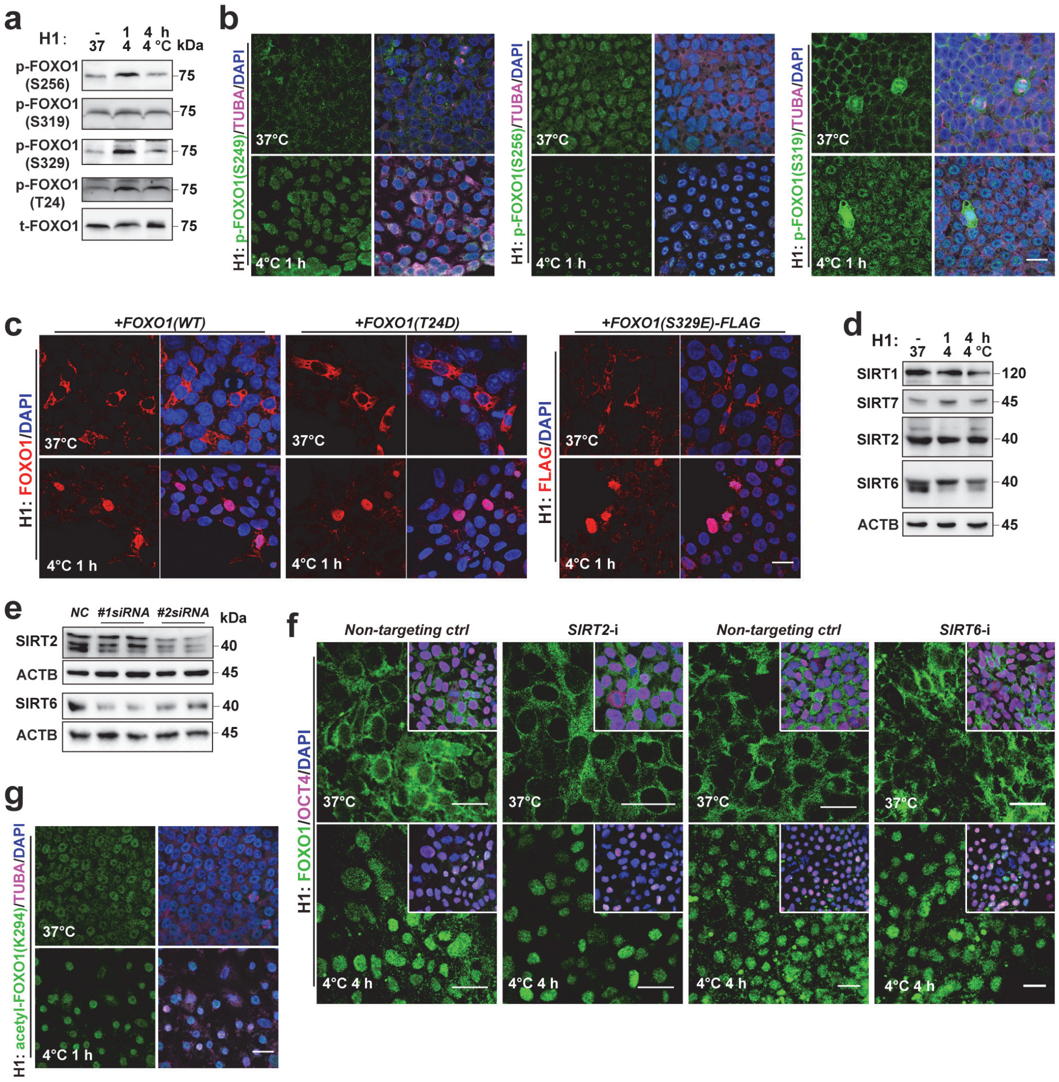

Supplementary Fig. 3 | Cold-induced FOXO1 nuclear entry independent of phosphorylation or Sirtuin-mediated modifications. a, Representative immunoblots of S256, S319, S329 and T24-phosphorylated form of FOXO1 proteins (p-FOXO1) in H1 cells at indicated conditions, revealing elevated levels of p-FOXO1 following 4°C incubation (n = 5 experiments). **b**, Confocal images of S249, S256 and S319 phosphorylated FOXO1, tubulin protein TUBA and DAPI staining in H1 cells at indicated conditions, revealing unexpected nuclear localization of p-FOXO1 proteins at 4°C (n = 5 experiments). **c**, Confocal images of FOXO1 and DAPI staining in H1 cells overexpressing *wild-type FOXO1* (*FOXO1(WT)*), or *FOXO1(T24D)* or *FOXO1(S329E)* mutant proteins that imitate a constitutively phosphorylated form of FOXO1 at indicated conditions, revealing cold-induced nuclear translocation of these overexpressed proteins (n = 5 experiments). **d**, Representative immunoblots of Sirtuin proteins (SIRT1, 2, 6 and 7 are shown as examples) in H1 cells at indicated conditions (n = 3 experiments). **e**, Representative immunoblots of SIRT2 and SIRT6 in *negative control* (*NC*) siRNA-, *SIRT2* or *SIRT6* siRNA-treated H1 cells (n = 3 experiments). **f**, Confocal images of FOXO1, pluripotency marker OCT4 and DAPI staining in H1

cells at indicated conditions, revealing that repressing the function of SIRT2 or SIRT6 did not overtly affect the temperature-induced FOXO1 translocation in H1 cells ($n = 3$ experiments). **g**, Confocal images of K294 acetyl-FOXO1, TUBA and DAPI staining in H1 cells at indicated conditions, revealing nuclear localization of acetyl-FOXO1 proteins at 4°C ($n = 5$ experiments). Scale bars: 20 μm (**b**, **c**, **f** and **g**). Source data are provided as a Source Data file.

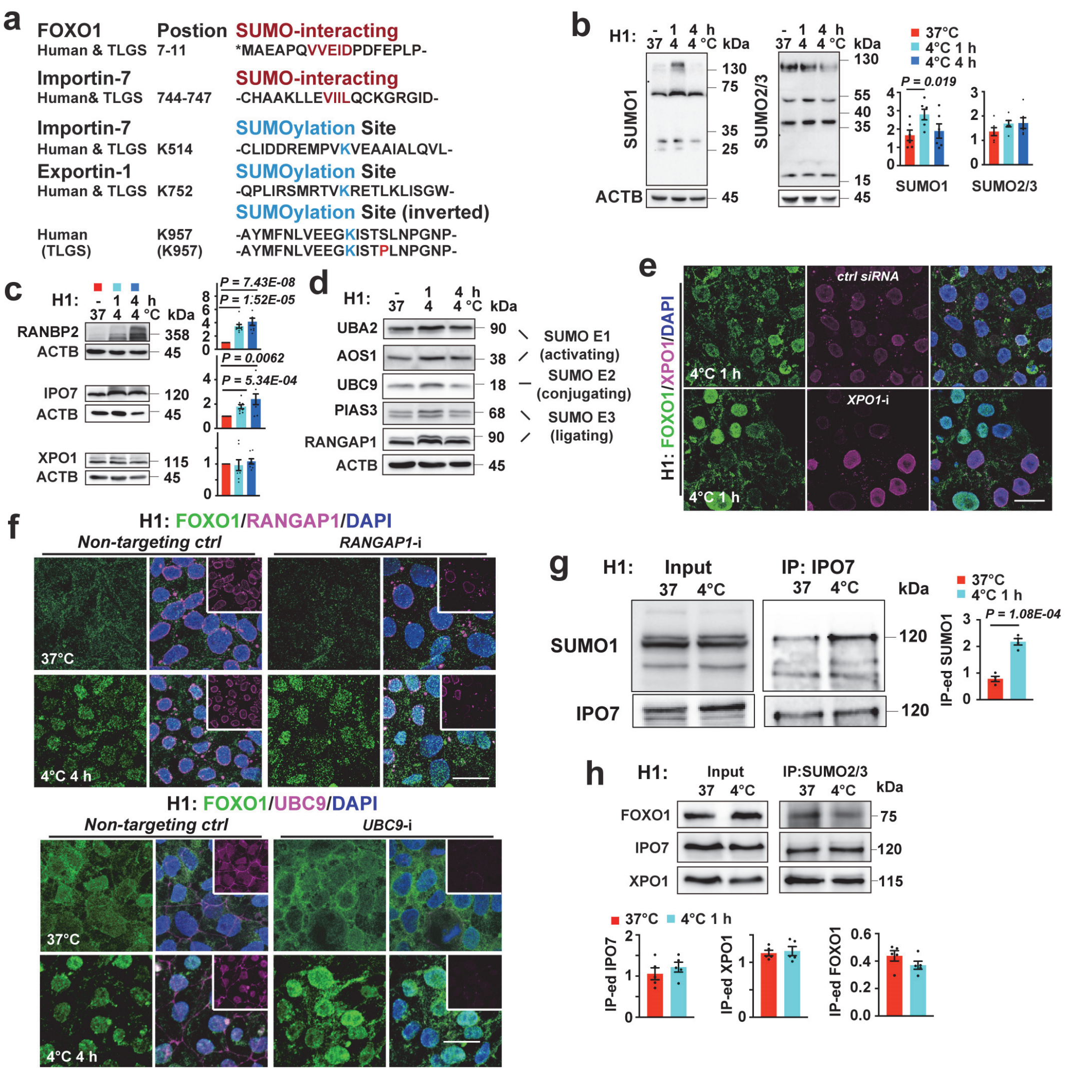

Supplementary Fig. 4 | FOXO1 and the SUMO machinery in cold adaptation. a, Human and TLGS FOXO1, Importin-7 and Exportin-1 are predicted to contain highly conserved SUMO-interacting motifs or SUMOylation sites. **b**, Left: representative immunoblots of SUMOylated proteins and free SUMO proteins in H1 cells at indicated conditions; right: normalized intensity levels of all SUMOylated proteins (n = 6 experiments). **c**, Left: representative immunoblots of RANBP2, IPO7 and XPO1 in H1 cells at annotated conditions; right: normalized intensity levels (n = 10, 8 and 8 experiments for RANBP2, IPO7 and XPO1, respectively). **d**, Representative immunoblots of some key components of SUMO ligases in H1 cells at indicated conditions (n = 5 experiments). **e**, Confocal images of FOXO1, XPO1 and DAPI staining in H1 ESCs at indicated conditions (n = 5 experiments). **f**, Confocal images of FOXO1, DAPI and RANGAP1 (up) or UBC9 (down) staining in H1 cells at indicated conditions, revealing that repressing the function of SUMO E3 ligase subunit RANGAP1 or UBC9 did not overtly affect the temperature-induced FOXO1 translocation in H1 cells (n = 3 experiments). **g**, Left: immunoblots of SUMO1 in total protein extracts (Input) and IPO7 IP-ed fractions from H1 cells at annotated conditions; right: signal intensities normalized (n = 4 experiments). **h**, Up:

immunoblots of XPO1, IPO7 and FOXO1 in Input and SUMO2/3 IP-ed fractions from H1 cells at annotated conditions; down: signal intensities normalized (n = 5 experiments). Data are shown as mean and SEM. Statistics: two-tailed Student's *t*-test (**b**, **c** and **g**). Scale bars: 20 µm (**e** and **f**). Source data are provided as a Source Data file.

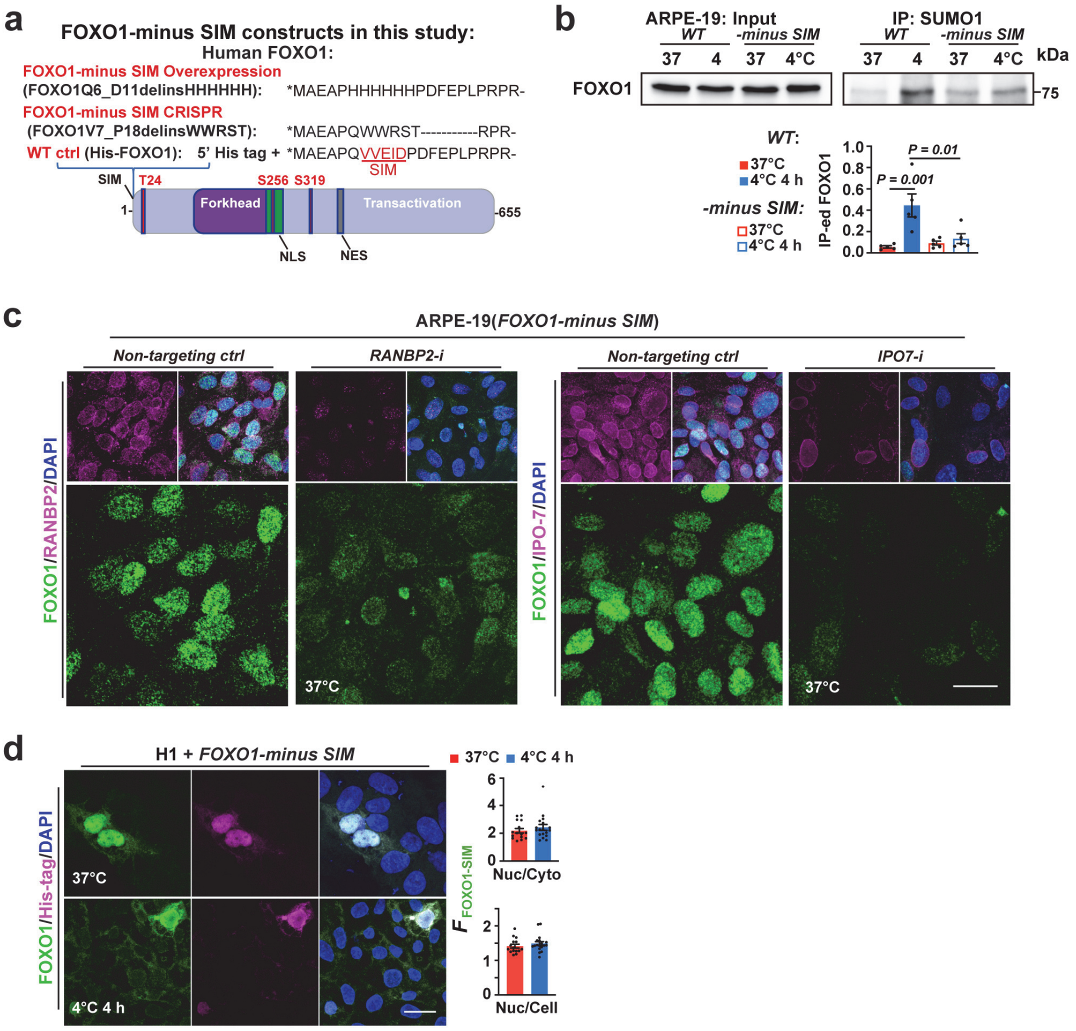

Supplementary Fig. 5 | Regulation of FOXO1 cellular distribution by FOXO1 SIM. **a**, Schema of FOXO1-minus SIM mutant constructs used in this study; NLS, nuclear localization sequence that is recognized by Importins; NES, nuclear export signal that is recognized by Exportins. **b**, Up: immunoblots of FOXO1 in Input and SUMO1 IP-ed fractions from ARPE-19 cells at annotated conditions; down: signal intensities normalized (n = 5 experiments). **c**, Confocal images of FOXO1, DAPI and RANBP2 (left) or Importin-7 (right) staining in ARPE-19(*FOXO1-minus SIM*) cells at indicated conditions (n = 5 experiments). **d**, Left: confocal images of FOXO1, His-tag and DAPI in H1 cells overexpressing *FOXO1-minus SIM* at indicated conditions; right: F_{FOXO1} ratio analyzed (from left to right, n = 15 and 18 images from 5 experiments). Data are shown as mean and SEM. Statistics: one-way ANOVA followed by Tukey's test (**b**). Scale bars: 20 µm (**c** and **d**). Source data are provided as a Source Data file.

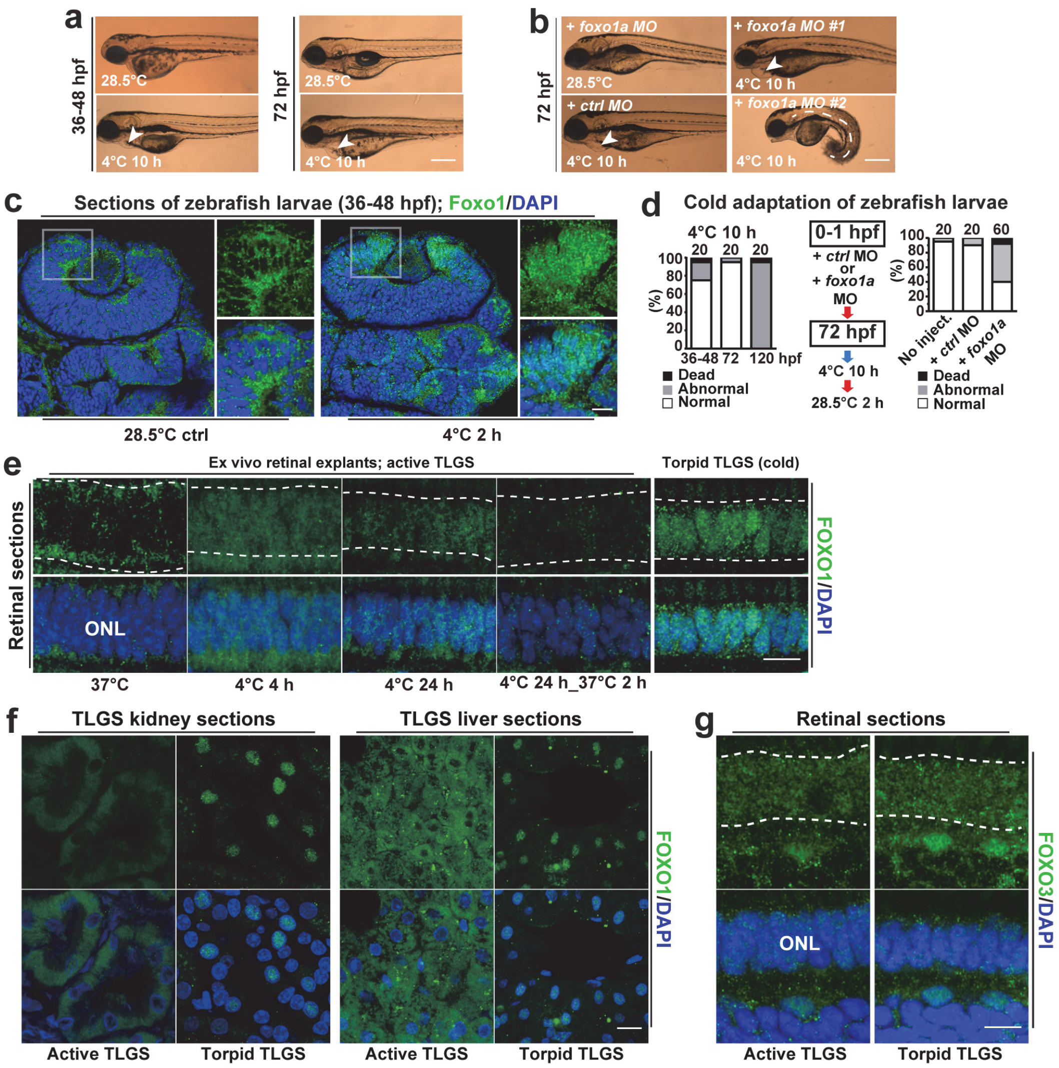

Supplementary Fig. 6 | The nuclear transport-FOXO1 axis and cold adaptation in zebrafish and 13-lined ground squirrel (TLGS). a, The morphology of zebrafish larvae at indicated conditions (n = 20); arrowheads indicate cold-induced pericardiac edema that are often reversible; hpf, hours post-fertilization. **b**, The morphology of zebrafish larvae with morpholino (MO; used in zebrafish to interfere with the expression of targeted genes) injection and 4°C incubation as indicated (n = 20 and 60 for *ctrl* and *foxo1a*, respectively); arrowheads indicate cold-induced pericardiac edema; broken line indicates irreversible cold-induced body curvatures. **c**, Confocal images of FOXO1 and DAPI in sections of zebrafish larvae at annotated conditions (n = 6). **d**, Summary on cold-induced abnormalities (see also Movie S1) and fatality in zebrafish larvae at annotated conditions; the numbers of larvae tested are at the top of the graphs. **e**, Confocal images of FOXO1 and DAPI staining in TLGS retinas from indicated conditions; broken lines highlight the outer nuclear layer (ONL) of the TLGS retinas (n = 8 in each group). **f**, Confocal images of FOXO1 and DAPI in TLGS kidneys and livers from indicated conditions (n = 8 in each group). **g**, Confocal

images of FOXO3 and DAPI in TLGS retinas from indicated conditions (n = 8 in each group). Scale bars: 20 µm (**c** and **e**-**g**) and 500 µm (**a** and **b**). Source data are provided as a Source Data file.

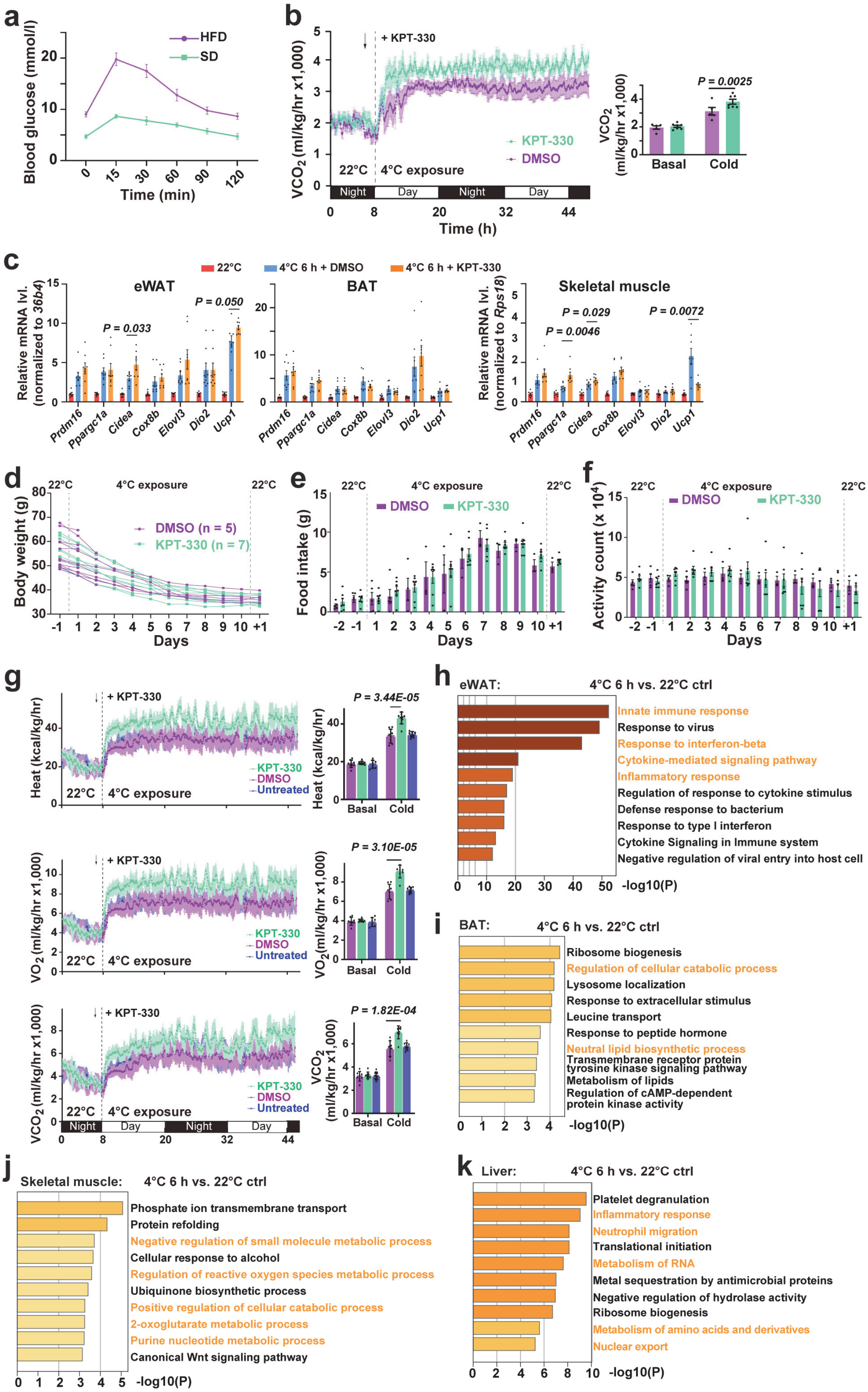
a
Blood glucose (mmol/l)
Time (min)
HFD
SD
b
VCO2 (ml/kg/hr x1,000)
+ KPT-330
22°C
4°C exposure
KPT-330
DMSO
Night
Day
Time (h)
P = 0.0025
Basal
Cold
c
22°C
4°C 6 h + DMSO
4°C 6 h + KPT-330
eWAT
BAT
Skeletal muscle
Relative mRNA lvl. (normalized to 36b4)
Relative mRNA lvl. (normalized to Rps18)
P = 0.050
P = 0.033
P = 0.029
P = 0.0046
P = 0.0072
Prdm16
Ppargc1a
Cidea
Cox8b
Elovl3
Dio2
Ucp1
d
Body weight (g)
DMSO (n = 5)
KPT-330 (n = 7)
Days
e
Food intake (g)
f
Activity count (x 10^4)
g
Heat (kcal/kg/hr)
VO2 (ml/kg/hr x1,000)
VCO2 (ml/kg/hr x1,000)
Untreated
P = 3.44E-05
P = 3.10E-05
P = 1.82E-04
h
eWAT:
4°C 6 h vs. 22°C ctrl
Innate immune response
Response to virus
Response to interferon-beta
Cytokine-mediated signaling pathway
Inflammatory response
Regulation of response to cytokine stimulus
Defense response to bacterium
Response to type I interferon
Cytokine Signaling in Immune system
Negative regulation of viral entry into host cell
-log10(P)
i
BAT:
Ribosome biogenesis
Regulation of cellular catabolic process
Lysosome localization
Response to extracellular stimulus
Leucine transport
Response to peptide hormone
Neutral lipid biosynthetic process
Transmembrane receptor protein tyrosine kinase signaling pathway
Metabolism of lipids
Regulation of cAMP-dependent protein kinase activity
j
Skeletal muscle:
Phosphate ion transmembrane transport
Protein refolding
Negative regulation of small molecule metabolic process
Cellular response to alcohol
Regulation of reactive oxygen species metabolic process
Ubiquinone biosynthetic process
Positive regulation of cellular catabolic process
2-oxoglutarate metabolic process
Purine nucleotide metabolic process
Canonical Wnt signaling pathway
k
Liver:
Platelet degranulation
Inflammatory response
Neutrophil migration
Translational initiation
Metabolism of RNA
Metal sequestration by antimicrobial proteins
Negative regulation of hydrolase activity
Ribosome biogenesis
Metabolism of amino acids and derivatives
Nuclear export

Supplementary Fig. 7 | KPT-330 activating FOXO1 nuclear entry to enhance cold survival in obese mice. a, Glucose tolerance test (GTT) after 3 months of high fat diet (HFD) or standard diet (SD) exposure by using an intraperitoneal injection (i.p.) dose of 1.5 g of glucose per kg of body weight (n_{HFD} = 11, n_{SD} = 4), suggesting pre-diabetic symptoms in mice with HFD. **b**, Left: whole-body CO_2 production (VCO_2) in obese mice with an i.p. of KPT-330 (3 mg/kg) (n = 7) or DMSO (n = 5) prior to 4°C exposure; right: average VCO_2 at basal and cold-exposed conditions. **c**, Quantitative PCR assays on the expression of designated thermogenic genes in epididymal white adipose tissue (eWAT), brown adipose tissue (BAT) and skeletal muscle samples from obese mice (12-14 month) at annotated conditions (n = 8 for each group). **d**-**f**, Body weight change (KPT-330, n = 9; DMSO, n = 5) (**d**), total food intake (KPT-330, n = 7; DMSO, n = 5) (**e**) and locomotor activities (KPT-330, n = 7; DMSO, n = 5) (**f**) of obese mice at annotated conditions. **g,** Left: Whole-body heat production, oxygen consumption (VO_2) and carbon dioxide production (VCO_2) in young healthy adult mice with no treatment, or with an i.p. of KPT-330 (3 mg/kg) or DMSO (n = 8 in each group) prior to 4°C exposure; right: average heat production, VO_2 and VCO_2 at basal and cold-exposed conditions. **h**-**k**, Enrichment analysis on DEGs upregulated in eWAT (**h**), BAT (**i**), skeletal muscles (**j**) and liver (**k**) at indicated conditions. Data are shown as mean and SEM. Statistics: two-tailed Student's *t*-test (**b**, **c** and **g**). *P* values in (**h**-**k**) were calculated in Metascape. Source data are provided as a Source Data file.

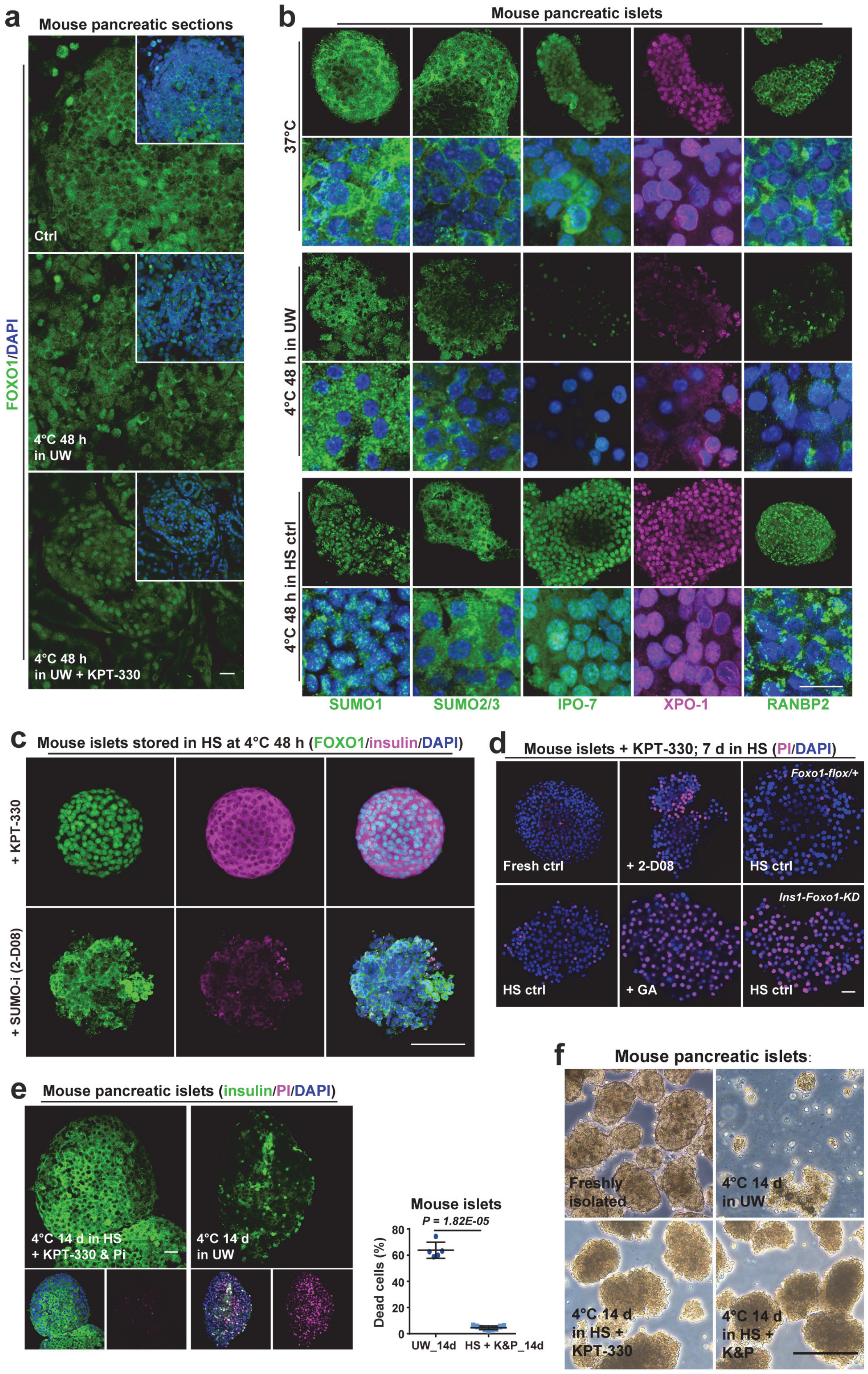

Supplementary Fig. 8 | Static cold storage of mouse pancreases and islets. a, Confocal images of FOXO1 and DAPI staining in mouse pancreatic sections collected

from indicated conditions (n = 10 mice); UW, the University of Wisconsin solution. **b**, Confocal images of DAPI and SUMO1, SUMO2/3, PO7, XPO1 or RANBP2 staining in isolated mouse islets at indicated conditions (n = 6 experiments); HS, basal hibernation solution used in cold exposure experiments. **c**, Confocal images of FOXO1, Insulin and DAPI staining in mouse islets at indicated conditions (n = 3 experiments); SUMO-i (2-D08), SUMOylation inhibitor. **d**, Confocal images of PI and DAPI staining in mouse islets at indicated conditions (n = 3 experiments); GA, Ginkgolic acid, SUMOylation inhibitor; *Ins1-Foxo1-KD*, inducible pancreatic β cell-specific knock-down of *Foxo1*; *Foxo1-flox/+*, mice with the *Foxo1-flox* knock-in allele but without the *Ins1-CreERT2* knock-in allele. **e**, Left: confocal images of insulin, PI and DAPI staining of mouse islets at indicated conditions; right: percentage of dead cells in mouse islets cold-stored in UW or HS + KPT-330 and protease inhibitors (Pi) for 14 days (n = 5 and 9 images from 5 experiments, respectively); K&P, KPT-330 and Pi. **f**, The morphology of mouse islets, treated as indicated and rewarmed to 37°C for 2 h; note the severe disintegration and autolysis of islets stored in UW solution for 14 d (n = 6 experiments). Data are shown as mean and SEM. Statistics: two-tailed Student's *t*-test (**e**). Scale bars: 20 μm (**a**, **b** and **e**), and 100 μm (**c**). Source data are provided as a Source Data file.

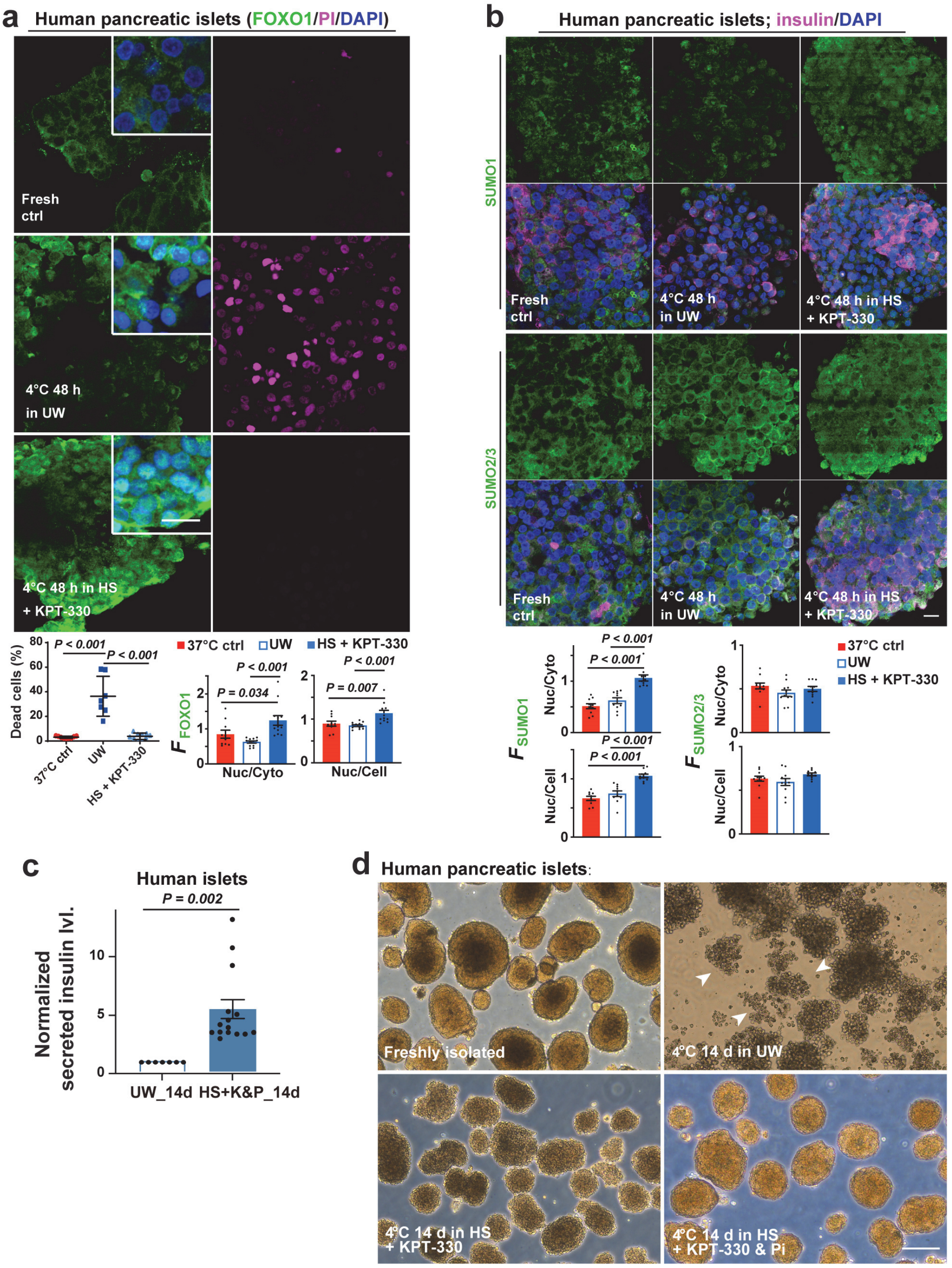

Supplementary Fig. 9 | Static cold storage of human pancreatic tissues and islets. **a**, Up: confocal images of dissociated human pancreatic islets at annotated conditions stained with FOXO1, PI and DAPI; down: dead cell percentage (n = 9, 7 and 10 images from 3 donors in ctrl, UW and HS + KPT-330 groups, respectively) and F_{FOXO1} ratio (n = 10, 12 and 12 images from 3 donors in ctrl, UW and HS + KPT-330 groups, respectively); UW, the University of Wisconsin solution; HS, basal hibernation solution used in cold exposure experiments. **b**, Up: confocal images of

insulin, SUMO1 or SUMO2/3, and DAPI staining in human pancreatic islets at indicated conditions; down: F_{SUMO1} (n = 10 images from 3 donors in ctrl, UW and HS + KPT-330 groups, respectively) and $F_{\mathrm{SUMO2/3}}$ (n = 11, 10 and 11 images from 3 donors in ctrl, UW and HS + KPT-330 groups, respectively) ratio analyzed. **c**, Normalized levels of secreted insulin in human islets at annotated storage conditions (n = 7 and 15 islet samples from 7 donors in UW and K&P groups, respectively); K&P, KPT-330 and protease inhibitors. **d**, The morphology of pancreatic islets isolated from donor pancreatic samples, treated as indicated and rewarmed to 37°C for 2 h; note the disintegration of cell mass from islets (arrowheads) stored in UW solution for 14 d (n = 3 experiments). Data are shown as mean and SEM. Statistics: one-way ANOVA followed by Tukey's test (**a** and **b**) or two-tailed Student's *t*-test (**c**). Scale bars: 20 μm (**b** and **c**) and 200 μm (**d**). Source data are provided as a Source Data file.

Supplementary Table 1.

Donor	Gender	Cause of Death	Previous condition
1	Female	Stroke	Hypertension
2	Male	Stroke	Not reported
3	Male	Head trauma	Not reported
4	Male	Brain aneurysm	Hypertension
5	Female	Stroke	Not reported
6	Male	Brain aneurysm	Hypertension
7	Female	Stroke	Hypertension
8	Male	Anoxia	Not reported
9	Male	Head trauma	Not reported

Pancreas donor information.

Supplementary Table 2.

TLGS iPSC Medium	Supplier	Catalog #	Concentration
DMEM/F12	Stem cell	36254	-
Knockout Serum Replacement	Thermo Fisher	A3181502	15%
Non-Essential Amino Acids	Thermo Fisher	11140-050	1x
L-Glutamine (200mM)	Thermo Fisher	25030-081	2mM
L-Ascorbic acid (50 mM in PBS)	MCE	HY-B0166	200 µM
b-FGF	R&D Systems	233-FB-025	40ng/mL

Human NPC medium	Supplier	Catalog #	Concentration
DMEM	Thermo Fisher	C11995500BT	-
B27 supplement (50x), no VitA	Thermo Fisher	12587010	1x
L-Glutamine (200mM)	Thermo Fisher	25030-081	2mM
b-FGF	R&D Systems	233-FB-025	20ng/mL
Human NOGGIN	R&D Systems	6057-NG	10ng/mL
Human EGF	R&D Systems	236-EG	20ng/mL

GS NPC medium	Supplier	Catalog #	Concentration
DMEM	Thermo Fisher	C11995500BT	-
B27 supplement (50x), no VitA	Thermo Fisher	12587010	1x
L-Glutamine (200mM)	Thermo Fisher	25030-081	2mM
b-FGF	R&D Systems	233-FB-025	20ng/mL
Human NOGGIN	R&D Systems	6057-NG	10ng/mL
Human EGF	R&D Systems	236-EG	20ng/mL
Knockout Serum Replacement	Thermo Fisher	A3181502	5% volume

Human ND medium	Supplier	Catalog #	Concentration
Neurobasal-A medium	Thermo Fisher	10888022	-
L-Glutamine (200mM)	Thermo Fisher	25030-081	2mM
B27 supplement (50x)	Thermo Fisher	17504044	1x
BDNF	R&D Systems	248-BD	10ng/mL
GDNF	R&D Systems	212-GD	2.5ng/mL

TLGS ND1 medium	Supplier	Catalog #	Concentration
DMEM	Thermo Fisher	C11995500BT	-
L-Glutamine (200mM)	Thermo Fisher	25030-081	2mM
B27 supplement (50x)	Thermo Fisher	17504044	1x
BDNF	R&D Systems	248-BD	10ng/mL
GDNF	R&D Systems	212-GD	2.5ng/mL
Knockout Serum Replacement	Thermo Fisher	A3181502	5% volume

Activin A	R&D Systems	338-AC	20ng/mL
**TLGS ND2 medium**	**Supplier**	**Catalog #**	**Concentration**
ND1 minus Activin A	-	-	-
**TLGS ND3 medium**	**Supplier**	**Catalog #**	**Concentration**
ND2 minus Knockout Serum	-	-	-

Hibernation solution (HS)	Supplier	Catalog #	Concentration
Hibernate-A medium	Thermo Fisher	A12475-01	Adjust osmolality to 305-320 mOsm/kg

University of Wisconsin Solution (Osmolality: 305-320 mOsm/kg)	Supplier	Catalog #	Concentration
Hydroxyethyl starch (Pentafraction)	Sigma	H6382	50 g/L
Lactobionic acid (as Lactone)	Sigma	L2398	35.83 g/L
Potassium dihydrogen phosphate	Sigma	P5655	3.4 g/L
Magnesium sulfate heptahydrate	Sigma	63138	1.23 g/L
Raffinose pentahydrate	Sigma	R0250	17.83 g/L
Adenosine	Sigma	A4036	1.34 g/L
Allopurinol	Sigma	A8003	0.136 g/L
Total Glutathione	Sigma	1294820	0.922 g/L
Potassium hydroxide	Sigma	1050291000	5.61 g/L
Sodium hydroxide	Sigma	S8045	Adjust pH 7.4
Hydrochloric acid	Sigma	H1758	Adjust pH 7.4

Human ESC medium	Supplier	Catalog #	Concentration
mTeSR Plus Kit	Stem Cell	05825	-

Human ARPE-19 medium	Supplier	Catalog #	Concentration
DMEM	Thermo Fisher	C11995500BT	-
Fetal Bovine Serum	Thermo Fisher	10099141	10%
Antibiotic Antimycotic	Thermo Fisher	15240062	1x

Other reagents	Supplier	Catalog #	Concentration
RPMI 1640	Gibco	C11875500BT	-
Geltrex Matrix	Thermo Fisher	A1569601	1x
Matrigel	Corning	354277	4%
Poly-L-ornithine	Sigma		0.01%
Laminin	R&D Systems	3446-005-01	5 µg/ml
Antibiotic Antimycotic	Thermo Fisher	15240062	1x

mFreSR™ Serum-free cryopreservation medium	Stem Cell	05855	-
Trypsin-EDTA (0.25%)	Thermo Fisher	25200-056	-
StemPro™ Accutase™ Cell Dissociation Reagent	Thermo Fisher	A1110501	-
Y-27632	MCE	HY-10071	-
EDTA	Thermo Fisher	15575-038	0.1%
AS1842856 (FOXO1 inhibitor)	Tocris/Biotechne	4265	2-10μM
Ginkgolic Acid (SUMOylation inhibitor)	MCE	HY-N0077	10μM
2-D08 (SUMOylation inhibitor)	MCE	HY-114166	10μM
TRIzol	Thermo Fisher	15596-026	-
Lipofectamin 3000	Thermo Fisher	L3000008	-
siRNA transfection reagent	Ribobio	C10511-1	-
Streptozotocin	Sigma	S0130	150 mg/kg
TUNEL assay	Roche	12156792910	-
Collagenase P	Roche	11213865001	Mouse: 0.5 mg/ml<br>Human: 2.5 mg/ml
Histopaque 1077	Sigma	RNBJ5120	
KRBH buffer	Phygene	PH1832-D	
KPT-330	Selleck	S7252	Mouse: 3 mg/kg<br>Pancreas/islets: 1μM
Pepstatin	Sigma-Aldrich	P5318	20μM
Leupeptin	Roche	11017128001	20μM
Mammalian protein extraction Reagent	Thermo Fisher	78501	-
Tissue protein extraction Reagent	Thermo Fisher	78510	-
Human insulin ELISA	Mercodia	10-1113-96	-
Mouse insulin ELISA	Mercodia	10-1247-01	-

Mediums, solutions and other reagents

Supplementary Table 3.

**Plasmids**	**5' sequencing primer**	**3' sequencing primer**
pcDNA3.1-*CMV-His-FOXO1*	CGCAAATGGGCGGTAGGCGTG	TAGAAGGCACAGTCGAGG
pcDNA3.1-*CMV-FOXO1SIMdelinsHis*	CGCAAATGGGCGGTAGGCGTG	TAGAAGGCACAGTCGAGG
pGV141-*CMV-FOXO1K559R-3XFLAG*	CGCAAATGGGCGGTAGGCGTG	TAGAAGGCACAGTCGAGG
pEZ-*CMV-FOXO1-3XFLAG*	GCGGTAGGCGTGTACGGT	CTGGAATAGCTCAGAGGC
pEZ-*CMV-FOXO1-minus NLS/NES-3XFLAG*	GCGGTAGGCGTGTACGGT	CTGGAATAGCTCAGAGGC
pEZ-*CMV-FOXO1(S329E)-3XFLAG*	GCGGTAGGCGTGTACGGT	CTGGAATAGCTCAGAGGC
pEZ-*CMV-FOXO3-3XHA*	GCGGTAGGCGTGTACGGT	GTGGCACCTTCCAGGGTC
pEZ-*CMV-FOXO3A6_L14delinsQVVEIDPDF-3XHA*	GCGGTAGGCGTGTACGGT	GTGGCACCTTCCAGGGTC
pTK-PCDH-copGFP-T2A-Puro-*CRM1(K752R)(K957R)-HA*	TGGGAGGTCTATATAAGCAGAG	GCCAGTACACGACATCACTT
pTK-PCDH-copGFP-T2A-Puro-*IPO7(K514R)-HA*	TGGGAGGTCTATATAAGCAGAG	GCCAGTACACGACATCACTT
pTK-PCDH-copGFP-T2A-Puro-*FOXO1(T24D)-HA*	CGCAAATGGGCGGTAGGCGTG	TTTCGCCCTAACTTCGTGAT
pTK-PCDH-copGFP-T2A-Puro- *FOXO1-HA*	CGCAAATGGGCGGTAGGCGTG	TTTCGCCCTAACTTCGTGAT

**qPCR target genes**	**5' primer**	**3' primer**
UCP1	CTTTGCCTCACTCAGGATTGG	ACTGCCACACCTCCAGTCATT
Ppargc1a	AGCCGTGACCACTGACAACGAG	GCTGCATGGTTCTGAGTGCTAAG
Cidea	ATCACAACTGGCCTGGTTACG	TACTACCCGGTGTCCATTTCT
Dio2	CAGCTTCCTCCTAGATGCCTA	GCTGAACCAAAGTTGACCACC
Elovl3	TCCGCGTTCTCATGTAGGTCT	GGACCTGATGCAACCCTATGA
Cox8b	GAACCATGAAGCCAACGACT	GCGAAGTTCACAGTGGTTCC
Prdm16	GGCGAGGAAGCTAGCCAAA	GGTCTCCTCCTCGGCACTCT
36B4	GCTTCGTGTTCACCAAGGAGGA	GTCCTAGACCAGTGTTCTGAGC
Rps18	CGCCATGTCTCTAGTGATCC	GGTCGATGTCTGCTTTCCTC

**siRNA/morpholino (MO) target genes**	**Sequence**
SIRT2	GCGCGTTTCTTCTCCTGTA
SIRT6	AGTGTAAGACGCAGTACGTCC
RANBP2	CATGCCGGGTGAAGGATTA
UBC9	AGCAGAGGCCTACACGATT
RANGAP1	GAAACCGTCTGGAGAATGA

IPO7	GCAAGAAGACCCTTACGAA
XPO1	GGAACATGATCAACTTATA
Zebrafish Foxo1a MO1	TACCAGCAATACTGTCTGCCTATTA
Zebrafish Foxo1a MO2	TGAAATGATAAATACCTTCCAGCCC

Plasmids, qPCR primers and siRNAs

Supplementary Table 4.

Antibodies	Supplier	Catalog #	Concentration
FOXO1	Abcam	ab52857	IF 1:250; WB 1:1000
FOXO1	Cell Signaling Technologies	2880	IF 1:250; WB 1:1000
FOXO3	Cell Signaling Technologies	12829	IF 1:250; WB 1:1000
TUBB3	Cell Signaling Technologies	5666	IF 1:500
TUBA	Cell Signaling Technologies	3873	IF 1:500
ACTB	Cell Signaling Technologies	4970	WB 1:1000
Phospho-FOXO1 (Ser249)	Thermo Fisher	PA564676	IF 1:100
Phospho-FOXO1 (Ser319)	Thermo Fisher	PA537577	IF 1:100; WB 1:500
Phospho-FOXO1 (Ser256)	Thermo Fisher	PA5104977	IF: 1:100; WB 1:500
Phospho-FOXO1 (Ser329)	Thermo Fisher	BS13207R	WB 1:500
Phospho-FOXO1 (Thr24)	Cell Signaling Technologies	9464	WB 1:500
Acetyl-FOXO1 (Lys294)	Thermo Fisher	PA5104560	IF 1: 100
SIRT1	Abcam	Ab110304	WB 1:1000
SIRT1	Cell Signaling Technologies	9475	WB 1:1000
SIRT2	Proteintech	19655-I-AP	WB 1:1000
SIRT6	Cell Signaling Technologies	12486	WB 1:1000
SIRT7	Cell Signaling Technologies	5360	WB 1:1000
IMPORTIN7	Santa Cruz Biotechnology	sc-365231	IF 1:200; WB 1:200
EXPORTIN1	Santa Cruz Biotechnology	sc-74454	IF 1:200; WB 1:200
UBC9	Santa Cruz Biotechnology	sc-271057	WB 1:200
UBA2	Santa Cruz Biotechnology	sc-376305	WB 1:200
AOS1	Santa Cruz Biotechnology	sc-271592	WB 1:200
PIAS3	Santa Cruz Biotechnology	sc-46682	WB 1:200
SUMO1	Santa Cruz Biotechnology	sc-5308	IF 1:200; WB 1:200
SUMO2/3/4	Santa Cruz Biotechnology	SC-393144	IF 1:200; WB 1:200
RANGAP1	Santa Cruz Biotechnology	sc-28322	IF 1:200; WB 1:200
RANBP2	Santa Cruz Biotechnology	sc-74518	IF 1:200; WB 1:200
GLUCAGON	Santa Cruz Biotechnology	sc-514592	IF 1:50
HA-TAG	Santa Cruz Biotechnology	sc-7392	IF 1:200; WB 1:200
UBC9	Santa Cruz Biotechnology	sc-271057	IF 1:200; WB 1:200
INSULIN	Abcam	ab181547	IF 1:200
Ki67	Abcam	ab15580	IF 1:200
HIS-TAG	Abcam	ab18184	IF 1:400; WB 1:1000
OCT4	Abcam	ab184665	IF 1:400
FLAG-TAG	Sigma	F3165	IF 1:400
GAPDH	Cell Signaling Technologies	2118	WB 1:1000
LMNB1	Cell Signaling Technologies	13435	WB 1:1000

Primary antibodies

Supplementary Table 5.
The inter-residue pairs with stable interactions in Supplementary Fig. 6c.

Celsius	No.	Group	Paris No.	Pairs	Average+/- Standard Error
**SUMO1-FOXO1 N-terminus**					
4	1	hb-foxo1-sim-donor	1	H17-V81	2.97+/-0.21
4	2	hb-foxo1-sim-donor	2	G19-I83	3.12+/-0.41
4	3	hb-foxo1-sim-donor	3	H17-V80	4.62+/-0.30
4	4	hb-foxo1-sim-donor	4	G19-E82	4.62+/-0.33
4	5	hb-foxo1-sim-donor	5	G19-D84	5.75+/-0.51
4	6	hb-foxo1-sim-donor	6	F18-V81	5.77+/-0.37
4	7	hb-foxo1-sim-donor	7	H17-E82	5.78+/-0.25
4	8	hb-foxo1-sim-donor	8	G19-V81	6.41+/-0.28
4	9	hb-sumo1-donor	1	G19-V81	2.91+/-0.17
4	10	hb-sumo1-donor	2	F18-V81	4.55+/-0.17
4	11	hb-sumo1-donor	3	K28-D84	4.72+/-2.29
4	12	hb-sumo1-donor	4	K21-I83	4.84+/-1.22
4	13	hb-sumo1-donor	5	T24-P85	5.01+/-1.57
4	14	hb-sumo1-donor	6	T24-D84	5.09+/-1.39
4	15	hb-sumo1-donor	7	H17-Q79	5.09+/-0.99
4	16	hb-sumo1-donor	8	V20-I83	5.31+/-0.81
4	17	hb-sumo1-donor	9	V20-V81	5.44+/-0.29
4	18	hb-sumo1-donor	10	T24-I83	5.45+/-1.17
4	19	hb-sumo1-donor	11	Y3-E82	5.56+/-1.86
4	20	hb-sumo1-donor	12	Y3-E82	5.56+/-1.89
4	21	hb-sumo1-donor	13	K28-D86	5.74+/-3.40
4	22	hb-sumo1-donor	14	G19-V80	5.81+/-0.47
4	23	hb-sumo1-donor	15	K28-D86	5.91+/-3.40
4	24	hb-sumo1-donor	16	K28-P85	6.03+/-2.52
4	25	hb-sumo1-donor	17	F18-Q79	6.20+/-0.56
4	26	hb-sumo1-donor	18	K28-D84	6.21+/-2.36
4	27	hb-sumo1-donor	19	K28-D84	6.23+/-2.38
4	28	hb-sumo1-donor	20	K28-P85	6.31+/-2.06
4	29	hb-sumo1-donor	21	G19-E82	6.33+/-0.31
4	30	hb-sumo1-donor	22	T24-P85	6.40+/-1.59
4	31	hb-sumo1-donor	23	F18-V80	6.50+/-0.26
4	32	hy	1	F18-V81	4.58+/-0.42
4	33	hy	2	V20-I83	5.25+/-0.52
4	34	hy	3	F18-I83	5.90+/-0.38
4	35	hy	4	L29-I83	5.99+/-0.57

4	36	hy	5	I16-V81	6.42+/-0.66
4	37	ele	1	K28-D86	6.50+/-2.61
4	38	ele	2	K28-D84	6.66+/-1.57
4	39	ele	3	K21-D84	8.80+/-2.43
4	40	ele	4	K21-E82	9.21+/-1.66
4	41	ele	5	K27-D86	9.56+/-2.95
4	42	ele	6	K5-E82	10.42+/-1.34
4	43	ele	7	K21-D86	10.46+/-2.94
37	1	hb-foxo1-sim-donor	1	H17-V81	2.99+/-0.23
37	2	hb-foxo1-sim-donor	2	G19-I83	3.19+/-0.63
37	3	hb-foxo1-sim-donor	3	H17-V80	4.55+/-0.27
37	4	hb-foxo1-sim-donor	4	G19-E82	4.77+/-0.57
37	5	hb-foxo1-sim-donor	5	G19-D84	5.56+/-0.67
37	6	hb-foxo1-sim-donor	6	H17-E82	5.67+/-0.32
37	7	hb-foxo1-sim-donor	7	F18-V81	6.05+/-0.52
37	8	hb-sumo1-donor	1	G19-V81	2.98+/-0.39
37	9	hb-sumo1-donor	2	H17-Q79	4.43+/-1.18
37	10	hb-sumo1-donor	3	F18-V81	4.54+/-0.22
37	11	hb-sumo1-donor	4	K28-D84	4.67+/-2.66
37	12	hb-sumo1-donor	5	K21-I83	5.11+/-1.40
37	13	hb-sumo1-donor	6	T24-D84	5.29+/-1.60
37	14	hb-sumo1-donor	7	T24-P85	5.48+/-1.98
37	15	hb-sumo1-donor	8	V20-I83	5.52+/-0.99
37	16	hb-sumo1-donor	9	V20-V81	5.56+/-0.48
37	17	hb-sumo1-donor	10	K28-P85	5.72+/-2.96
37	18	hb-sumo1-donor	11	K28-D84	5.77+/-2.45
37	19	hb-sumo1-donor	12	K28-D84	5.80+/-2.47
37	20	hb-sumo1-donor	13	T24-I83	5.84+/-1.23
37	21	hb-sumo1-donor	14	Y3-E82	5.93+/-2.13
37	22	hb-sumo1-donor	15	K28-P85	5.96+/-2.06
37	23	hb-sumo1-donor	16	Y3-E82	5.96+/-2.13
37	24	hb-sumo1-donor	17	F18-Q79	5.98+/-0.60
37	25	hb-sumo1-donor	18	G19-V80	6.24+/-0.80
37	26	hb-sumo1-donor	19	G19-E82	6.39+/-0.48
37	27	hb-sumo1-donor	20	T24-P85	6.49+/-2.06
37	28	hy	1	F18-V81	4.63+/-0.50
37	29	hy	2	V20-I83	5.21+/-0.52
37	30	hy	3	F18-I83	5.99+/-0.43
37	31	hy	4	L29-I83	6.14+/-0.57
37	32	hy	5	I16-V81	6.35+/-0.73
37	33	ele	1	K28-D84	6.39+/-1.67

37	34	ele	2	K28-D86	7.86+/-3.74
37	35	ele	3	K21-D84	8.52+/-2.41
37	36	ele	4	K21-D86	8.98+/-2.39
37	37	ele	5	K21-E82	9.74+/-1.85
37	38	ele	6	K5-E82	10.34+/-1.55
**SUMO1-FOXO3 N-terminus**					
4	1	hb-foxo3-notsim-donor	1	G19-A82	5.83+/-2.08
4	2	hb-foxo3-notsim-donor	2	H17-S80	6.12+/-2.37
4	3	hb-foxo3-notsim-donor	3	K28-L84	6.28+/-1.52
4	4	hb-sumo1-donor	1	G19-P81	5.25+/-2.89
4	5	hb-sumo1-donor	2	S32-P83	5.52+/-1.33
4	6	hb-sumo1-donor	3	G19-S80	5.65+/-2.64
4	7	hb-sumo1-donor	4	S32-A82	5.94+/-1.37
4	8	hb-sumo1-donor	5	K28-P83	6.01+/-1.83
4	9	hb-sumo1-donor	6	G19-P81	6.01+/-2.74
4	10	hb-sumo1-donor	7	S32-P83	6.24+/-1.85
4	11	hb-sumo1-donor	8	K28-L84	6.34+/-2.27
4	12	hy	1	F18-P83	6.47+/-3.03
37	1	hb-sumo1-donor	1	G19-P83	6.17+/-0.92
37	2	hb-sumo1-donor	2	S32-L84	6.35+/-2.28
37	3	hy	1	F18-P83	6.42+/-1.92
