# Supplementary material for: Cold-induced FOXO1 nuclear transport aids cold survival and tissue storage
Source: Nat Commun. 2024 Apr 3;15:2859. doi: 10.1038/s41467-024-47095-w (PMC10991392; doi:10.1038/s41467-024-47095-w)
Supplement: Supplementary file 3 — Description of Additional Supplementary Files [file 41467_2024_47095_MOESM3_ESM.pdf]

Description of Additional Supplementary Files

File Name: Supplementary Data 1
Description: Temperature-induced FOXO1-bound genes, differentially expressed genes and their common genes in H1 ESCs.

File Name: Supplementary Data 2
Description: Enrichment analysis on temperature-induced differentially expressed genes and common genes differentially bound by FOXO1 in H1 ESCs.

File Name: Supplementary Data 3
Description: Common differentially expressed genes (DEGs) in eWAT, BAT, skeletal muscle and liver of cold-exposed obese mice with or without KPT-330 treatment and enrichment analysis.

File Name: Supplementary Movie 1
Description: Zebrafish larvae with injection of control or *Foxo1a* morpholino recovering from 4°C exposure.
