# Supplementary material for: Cold-induced FOXO1 nuclear transport aids cold survival and tissue storage
Source: Nat Commun. 2024 Apr 3;15:2859. doi: 10.1038/s41467-024-47095-w (PMC10991392; doi:10.1038/s41467-024-47095-w)
Supplement: Supplementary file 8 — Reporting Summary [file 41467_2024_47095_MOESM8_ESM.pdf]

nature portfolio

Corresponding author(s): Wei Liu, Yang Yang, Wei Li, Jingxing Ou

Last updated by author(s): Feb 18, 2024

Reporting Summary

Nature Portfolio wishes to improve the reproducibility of the work that we publish. This form provides structure for consistency and transparency in reporting. For further information on Nature Portfolio policies, see our Editorial Policies and the Editorial Policy Checklist.

Statistics

For all statistical analyses, confirm that the following items are present in the figure legend, table legend, main text, or Methods section.

n/a	Confirmed	
☐	☒	The exact sample size (n) for each experimental group/condition, given as a discrete number and unit of measurement
☐	☒	A statement on whether measurements were taken from distinct samples or whether the same sample was measured repeatedly
☐	☒	The statistical test(s) used AND whether they are one- or two-sided<br>*Only common tests should be described solely by name; describe more complex techniques in the Methods section.*
☒	☐	A description of all covariates tested
☐	☒	A description of any assumptions or corrections, such as tests of normality and adjustment for multiple comparisons
☐	☒	A full description of the statistical parameters including central tendency (e.g. means) or other basic estimates (e.g. regression coefficient) AND variation (e.g. standard deviation) or associated estimates of uncertainty (e.g. confidence intervals)
☐	☒	For null hypothesis testing, the test statistic (e.g. F, t, r) with confidence intervals, effect sizes, degrees of freedom and P value noted<br>*Give P values as exact values whenever suitable.*
☒	☐	For Bayesian analysis, information on the choice of priors and Markov chain Monte Carlo settings
☒	☐	For hierarchical and complex designs, identification of the appropriate level for tests and full reporting of outcomes
☒	☐	Estimates of effect sizes (e.g. Cohen's d, Pearson's r), indicating how they were calculated

Our web collection on statistics for biologists contains articles on many of the points above.

Software and code

Policy information about availability of computer code

Data collection	Real-time PCR was performed using 7900HT Fast Real-Time PCR system. Confocal imaging was perform using the Zeiss LSM-880 or or by STED imaging (Leica, Wetzlar, Germany). RNA-seq Reads were aligned to the mouse genome (Ensembl GRCm38) or human genome (Ensembl GRCh38) with Hisat2 v2.0.5 and quantified with featureCounts v2.0.1. ATAC-seq and CUT&Tag: Fastp v0.21.0 was used to remove adapter and low-quality reads. Paired end reads were then mapped using Bowtie2 v2.4.2. After alignment, unique aligned reads were filtered with Picard MarkDuplicates v2.25.0. Normalized, fragment signal bigwigs were created using Deeptools v3.5.1, and visualization was performed with Integrative Genomics Viewer (IGV) software. Peaks were called individually for each replicate using macs2 v2.2.7.1
Data analysis	General data were analyzed using Graphpad Prism 7.0 or Microsoft Excel. Western blots were quantified with the ImageJ Gel Analysis tool. ATAC-seq: EdgeR embedded in Diffbind v2.14.0, Motif enrichment was performed with Homer v4.11.1; CUT&Tag: DESeq2 embedded in Diffbind v2.14.0; RNA-seq:DESeq2 v1.26.0. Pathway analysis: Metascape web interface.

For manuscripts utilizing custom algorithms or software that are central to the research but not yet described in published literature, software must be made available to editors and reviewers. We strongly encourage code deposition in a community repository (e.g. GitHub). See the Nature Portfolio guidelines for submitting code & software for further information.

Data

Policy information about availability of data

All manuscripts must include a data availability statement. This statement should provide the following information, where applicable:

- Accession codes, unique identifiers, or web links for publicly available datasets
- A description of any restrictions on data availability
- For clinical datasets or third party data, please ensure that the statement adheres to our policy

All sequencing data are available at NCBI GEO under accession number GSE185152. This paper does not report original code. Source data are provided with this paper.

Research involving human participants, their data, or biological material

Policy information about studies with human participants or human data. See also policy information about sex, gender (identity/presentation), and sexual orientation and race, ethnicity and racism.

Reporting on sex and gender	Gender of the donors of pancreatic tissues has been provided in Supplementary Data 1.
Reporting on race, ethnicity, or other socially relevant groupings	Donors in this study were all Chinese.
Population characteristics	Donor age range and other conditions are provided in Methods section and Supplementary Table 1.
Recruitment	Human pancreatic tissue was obtained from deceased patients consented to donate their organs for research. The use of human tissue samples complied with the Declaration of Helsinki and was approved by Institutional Ethics Committee of the Third Affiliated Hospital of Sun Yat-Sen University (No. 02-058-01).
Ethics oversight	Institutional Ethics Committee of the Third Affiliated Hospital of Sun Yat-Sen University.

Note that full information on the approval of the study protocol must also be provided in the manuscript.

Field-specific reporting

Please select the one below that is the best fit for your research. If you are not sure, read the appropriate sections before making your selection.

☒ Life sciences ☐ Behavioural & social sciences ☐ Ecological, evolutionary & environmental sciences

For a reference copy of the document with all sections, see nature.com/documents/nr-reporting-summary-flat.pdf

Life sciences study design

All studies must disclose on these points even when the disclosure is negative.

Sample size	No statistical method was use to predetermine sample sizes. The sample sizes used in this study were similar to those reported in previous publications (PMID: 32615086; 29576452; 25383517).
Data exclusions	Cell transfection experiments sometimes will cause abnormalities in morphology and physiology of the cultured cells, and hence the subsequent experimental results will be unreliable. In this case, the whole batch of cell cultures will be discarded, data will not be counted, and experiments will be repeated. Batch effects could also been seen in zebrafish larvae and obese mice, such as larvae size and morphology, and mouse bite wounds or dermatitis. Entities with notable defects will not be included into the experimental groups.
Replication	Experiments are repeated at least 3-5 times. Some experiments such as quantitative PCR would have 3 repeats of each sample. If visually apparent variation was seen in the repeats of a same sample, the results from this sample will not be counted, and new experimental repeat will be perform. Other situations are as stated above. All experiments were successfully repeated
Randomization	Dishes of cultured cells, zebrafish larvae, obese mice, mouse pancreases, donor pancreatic tissues, and mouse and human islets were randomly allocated to experimental groups.
Blinding	During experiments, the investigators were not blinded to allocation because the experiments must be performed, and data be collected by experienced investigators that knew the grouping and processed the information properly. In cases of image analyses on dead cell counting and PLA, junior investigators were blinded. Image capture and analysis were done by different investigators without knowing the grouping information.

Reporting for specific materials, systems and methods

We require information from authors about some types of materials, experimental systems and methods used in many studies. Here, indicate whether each material, system or method listed is relevant to your study. If you are not sure if a list item applies to your research, read the appropriate section before selecting a response.

Materials & experimental systems

n/a	Involved in the study
☐	☒ Antibodies
☐	☒ Eukaryotic cell lines
☒	☐ Palaeontology and archaeology
☐	☒ Animals and other organisms
☒	☐ Clinical data
☒	☐ Dual use research of concern
☒	☐ Plants

Methods

n/a	Involved in the study
☐	☒ ChIP-seq
☒	☐ Flow cytometry
☒	☐ MRI-based neuroimaging

Antibodies

Antibodies used

These commercially-available antibodies were used (also see Supplementary Table 4):
FOXO1 Abcam ab52857 IF 1:250; WB 1:1000
FOXO1 Cell Signaling Technologies 2880 IF 1:250; WB 1:1000
FOXO3 Cell Signaling Technologies 12829 IF 1:250; WB 1:1000
TUBB3 Cell Signaling Technologies 5666 IF 1:500
TUBA Cell Signaling Technologies 3873 IF 1:500
ACTB Cell Signaling Technologies 4970 WB 1:1000
Phospho-FOXO1 (Ser249) Thermo Fisher PA564676 IF 1:100
Phospho-FOXO1 (Ser319) Thermo Fisher PA537577 IF 1:100; WB 1:500
Phospho-FOXO1 (Ser256) Thermo Fisher PA5104977 WB 1:500
Phospho-FOXO1 (Ser329) Thermo Fisher BS13207R WB 1:500
Phospho-FOXO1 (Thr24) Cell Signaling Technologies 9464 WB 1:500
Acetyl-FOXO1 (Lys294) Thermo Fisher PA5104560 IF 1: 100
SIRT1 Abcam Ab110304 WB 1:1000
SIRT1 Cell Signaling Technologies 9475 WB 1:1000
SIRT2 Proteintech 19655-I-AP WB 1:1000
SIRT6 Cell Signaling Technologies 12486 WB 1:1000
SIRT7 Cell Signaling Technologies 5360 WB 1:1000
IMPORTIN7 Santa Cruz Biotechnology sc-365231 IF 1:200; WB 1:200
EXPORTIN1 Santa Cruz Biotechnology sc-74454 IF 1:200; WB 1:200
UBC9 Santa Cruz Biotechnology sc-271057 WB 1:200
UBA2 Santa Cruz Biotechnology sc-376305 WB 1:200
AOS1 Santa Cruz Biotechnology sc-271592 WB 1:200
PIAS3 Santa Cruz Biotechnology sc-46682 WB 1:200
SUMO1 Santa Cruz Biotechnology sc-5308 IF 1:200; WB 1:200
SUMO2/3/4 Santa Cruz Biotechnology SC-393144 IF 1:200; WB 1:200
RANGAP1 Santa Cruz Biotechnology sc-28322 IF 1:200; WB 1:200
RANBP2 Santa Cruz Biotechnology sc-74518 IF 1:200; WB 1:200
GLUCAGON Santa Cruz Biotechnology sc-514592 IF 1:50
HA-TAG Santa Cruz Biotechnology sc-7392 IF 1:200; WB 1:200
UBC9 Santa Cruz Biotechnology sc-271057 IF 1:200; WB 1:200
INSULIN Abcam ab181547 IF 1:200
Ki67 Abcam ab15580 IF 1:200
HIS-TAG Abcam ab18184 IF 1:400; WB 1:1000
OCT4 Abcam ab184665 IF 1:400
FLAG-TAG Sigma F3165 IF 1:400
GAPDH Cell Signaling Technologies 2118 WB 1:1000
LMNB1 Cell Signaling Technologies 13435 WB 1:1000

Validation

Validation statements and other antibody information:
FOXO1 Abcam ab52857 (https://www.abcam.com/products/primary-antibodies/foxo1a-antibody-ep927y-ab52857.html)
FOXO1 Cell Signaling Technologies 2880 (https://www.cellsignal.com/products/primary-antibodies/foxo1-c29h4-rabbit-mab/2880)
FOXO3 Cell Signaling Technologies 12829 (https://www.cellsignal.com/products/primary-antibodies/foxo3a-d19a7-rabbit-mab/12829)
TUBB3 Cell Signaling Technologies 5666 (https://www.cellsignal.com/products/primary-antibodies/b3-tubulin-d65a4-xp-rabbit-mab/5666)
TUBA Cell Signaling Technologies 3873 (https://www.cellsignal.com/products/primary-antibodies/a-tubulin-dm1a-mouse-mab/3873)
ACTB Cell Signaling Technologies 4970 (https://www.cellsignal.com/products/primary-antibodies/b-actin-13e5-rabbit-mab/4970)
Phospho-FOXO1 (Ser319) Thermo Fisher PA537577 (https://www.thermofisher.com/antibody/product/Phospho-FOXO1-Ser319-

Antibody-Polyclonal/PA5-37577)
Phospho-FOXO1 (Ser256) Thermo Fisher PA5104977 (https://www.thermofisher.com/antibody/product/Phospho-FOXO1-Ser256-Antibody-Polyclonal/PA5-104977)
Acetyl-FOXO1 (Lys294) Thermo Fisher PA5104560 (https://www.thermofisher.com/antibody/product/Acetyl-FOXO1-Lys294-Antibody-Polyclonal/PA5-104560)
Phospho-FOXO1 (Ser329) Thermo Fisher BS13207R (https://www.thermofisher.com/antibody/product/Phospho-FoxO1-Ser329-Antibody-Polyclonal/BS-13207R)
Phospho-FOXO1 (Ser249) (https://www.thermofisher.com/antibody/product/Phospho-FOXO1-Ser249-Antibody-Polyclonal/PA5-64676)
SIRT1 Abcam Ab110304 (https://www.abcam.co.jp/products/primary-antibodies/sirt1-antibody-19a7ab4-ab110304.html)
SIRT1 Cell Signaling Technologies 9475 (https://www.cellsignal.com/products/primary-antibodies/sirt1-d1d7-rabbit-mab/9475)
SIRT2 Proteintech 19655-I-AP (https://www.ptglab.com/products/SIRT2-Antibody-19655-1-AP.htm)
SIRT6 Cell Signaling Technologies 12486 (https://www.cellsignal.com/products/primary-antibodies/sirt6-d8d12-rabbit-mab/12486)
SIRT7 Cell Signaling Technologies 5360 (https://www.cellsignal.com/products/primary-antibodies/sirt7-d3k5a-rabbit-mab/5360)
IMPORTIN7 Santa Cruz Biotechnology sc-365231 (https://www.scbt.com/p/importin-7-antibody-e-2)
EXPORTIN1 Santa Cruz Biotechnology sc-74454 (https://www.scbt.com/p/crm1-antibody-c-1)
UBC9 Santa Cruz Biotechnology sc-271057 (https://www.scbt.com/ja/p/ubc9-antibody-c-12)
UBA2 Santa Cruz Biotechnology sc-376305 (https://www.scbt.com/ja/p/uba2-antibody-b-6)
AOS1 Santa Cruz Biotechnology sc-271592 (https://www.scbt.com/ja/p/aos-1-antibody-h-7)
PIAS3 Santa Cruz Biotechnology sc-46682 (https://www.scbt.com/ja/p/pias-3-antibody-c-12)
SUMO1 Santa Cruz Biotechnology sc-5308 (https://www.scbt.com/ja/p/sumo-1-antibody-d-11)
SUMO2/3/4 Santa Cruz Biotechnology SC-393144 (https://www.scbt.com/ja/p/sumo-2-3-4-antibody-c-3)
RANGAP1 Santa Cruz Biotechnology sc-28322 (https://www.scbt.com/ja/p/ran-gap1-antibody-c-5)
RANBP2 Santa Cruz Biotechnology sc-74518 (https://www.scbt.com/ja/p/ran-bp-2-antibody-d-4)
GLUCAGON Santa Cruz Biotechnology sc-514592 (https://www.scbt.com/ja/p/glucagon-antibody-c-11)
HA-TAG Santa Cruz Biotechnology sc-7392 (https://www.scbt.com/ja/p/ha-probe-antibody-f-7)
UBC9 Santa Cruz Biotechnology sc-271057 (https://www.scbt.com/ja/p/ubc9-antibody-c-12)
INSULIN Abcam ab181547 (https://www.abcam.com/products/primary-antibodies/insulin-antibody-epr17359-ab181547.html)
Ki67 Abcam ab15580 IF (https://www.abcam.com/products/primary-antibodies/ki67-antibody-ab15580.html)
HIS-TAG Abcam ab18184 (https://www.abcam.com/products/primary-antibodies/6x-his-tag-antibody-hish8-ab18184.html)
OCT4 Abcam ab184665 (https://www.abcam.com/products/primary-antibodies/oct4-antibody-gt486-ab184665.html)
FLAG-TAG Sigma F3165 (https://www.sigmaaldrich.com/TW/en/product/sigma/f3165)
GAPDH Cell Signaling Technologies 2118 (https://www.cellsignal.com/products/primary-antibodies/gapdh-14c10-rabbit-mab/2118)
LMNB1 Cell Signaling Technologies 13435 (https://www.cellsignal.com/products/primary-antibodies/lamin-b1-d9v6h-rabbit-mab/13435)

Eukaryotic cell lines

Policy information about cell lines and Sex and Gender in Research

Cell line source(s)	13-lined ground squirrel (TLGS) iPSCs and iPSC-neurons were established from P2 female TLGS pups and maintained as described previously (PMID: 29576452).<br>Human iPSC (1 female infant donor; 1 adult male donor)-neurons were cultured in the W.LI lab at the National Eye Institute following the NIH stem cell protocols (https://stemcells.nih.gov/research/nihresearch/scunit/protocols.htm).<br>H1 ESC line (male) was originally obtained from WiCell (Madison, USA).<br>Human retinal epithelial ARPE-19 cell line (established from a male donor) was obtained from the American Type Culture Collection (ATCC, Manassas, VA) .
Authentication	All cell lines were authenticated by STR analyses by a local service company.
Mycoplasma contamination	All cell lines were negative for mycoplasma in our monthly tests.
Commonly misidentified lines (See ICLAC register)	No commonly misidentified cell lines were used.

Animals and other research organisms

Policy information about studies involving animals; ARRIVE guidelines recommended for reporting animal research, and Sex and Gender in Research

Laboratory animals	The C57BL/6 (Gempharmatech, Nanjing, China), Ins1-IRES-iCreERT2 (Biocytogen, Beijing, China) and Foxo1-P2A-EGFP-flox (Gempharmatech, Nanjing, China) mice were bred at the animal facility of the Third Affiliated Hospital of Sun Yat-sen University in accordance with the requirements of the institutional Animal Review Board. For obese mice (12-14 month) (Gempharmatech, Nanjing, China), mice were switched to high-fat diet (OpenSource Diets, D12492) with 60% kcal from fat, 20% kcal from carbohydrate, and 20% kcal from protein for 3 months. All mice were housed with a 12/12 light/dark cycle at 20-25°C and 45-65% of relative humidity.<br>Zebrafish (Danio rerio) lines AB-wild type and Tg(myl7:EGFP) were acquired from China Zebrafish Resource Center and raised at 28± 0.5°C in 14 h light/10 h dark cycles.

Wild animals	This study did not involve wild animals.
Reporting on sex	Animals and tissues from donors of both male and female are treated as the same in this study. No overt gender differences were seen in all experiments.
Field-collected samples	This study did not involve samples collected from the field
Ethics oversight	All animal studies were in strict accordance with the recommendations in the Guide for the Care and Use of Laboratory Animals of the National Institutes of Health. All animals were handled according to approved institutional animal care and use committee (IACUC) protocols of the Third Affiliated Hospital of Sun Yat-sen University and China Zebrafish Resource Center.

Note that full information on the approval of the study protocol must also be provided in the manuscript.

Plants

Seed stocks	*Report on the source of all seed stocks or other plant material used. If applicable, state the seed stock centre and catalogue number. If plant specimens were collected from the field, describe the collection location, date and sampling procedures.*
Novel plant genotypes	*Describe the methods by which all novel plant genotypes were produced. This includes those generated by transgenic approaches, gene editing, chemical/radiation-based mutagenesis and hybridization. For transgenic lines, describe the transformation method, the number of independent lines analyzed and the generation upon which experiments were performed. For gene-edited lines, describe the editor used, the endogenous sequence targeted for editing, the targeting guide RNA sequence (if applicable) and how the editor was applied.*
Authentication	*Describe any authentication procedures for each seed stock used or novel genotype generated. Describe any experiments used to assess the effect of a mutation and, where applicable, how potential secondary effects (e.g. second site T-DNA insertions, mosiacism, off-target gene editing) were examined.*

ChIP-seq

Data deposition

☒ Confirm that both raw and final processed data have been deposited in a public database such as GEO.

☒ Confirm that you have deposited or provided access to graph files (e.g. BED files) for the called peaks.

Data access links<br>*May remain private before publication.*	All sequencing data are available at NCBI GEO under accession number GSE185152. Token to access GSE185152 is: svojkoachrglnej.
Files in database submission	Files are available in: GSE183998_RAW.tar
Genome browser session<br>(e.g. UCSC)	No longer applicable.

Methodology

Replicates	Two biological replicates were performed. All CUT&Tag data are derived from the analysis of both replicates.
Sequencing depth	Paired end reads were mapped to human genome (Ensembl GRCh38) using Bowtie2 v2.4.2 , and the proportion of uniquely mapped reads meet the standard quality control requirements.
Antibodies	FOXO1 Cell Signaling Technologies 2880 (https://www.cellsignal.com/products/primary-antibodies/foxo1-c29h4-rabbit-mab/2880)
Peak calling parameters	Peaks were called individually for each replicate using macs2 v2.2.7.112 (options: -f BAMPE --call-summits), with q=0.1 as the threshold for for CUT&Tag peaks.
Data quality	Fastp v0.21.0 was used to remove adapter and low-quality reads.
Software	Differential analysis of FOXO1-binding peaks in CUT&TAG were performed using DESeq2 embedded in Diffbind v2.14.0. Peaks with an FDR-corrected Q-value <0.05 were assigned as differentially expressed.
